# Inter- and Intra-Host Viral Diversity in a Large Seasonal DENV2 Outbreak

**DOI:** 10.1371/journal.pone.0070318

**Published:** 2013-08-02

**Authors:** Camila Malta Romano, Michael Lauck, Felipe S. Salvador, Célia Rodrigues Lima, Lucy S. Villas-Boas, Evaldo Stanislau A. Araújo, José Eduardo Levi, Claudio Sergio Pannuti, David O’Connor, Esper Georges Kallas

**Affiliations:** 1 Instituto de Medicina Tropical de São Paulo e Faculdade de Medicina, Departamento de Moléstias Infecciosas e Parasitárias (LIMHC), Universidade de São Paulo, São Paulo, Brazil; 2 Department of Pathology and Laboratory Medicine, University of Wisconsin–Madison, Madison, Wisconsin, United States of America; 3 Hospital das Clínicas da Faculdade de Medicina, Universidade de São Paulo, São Paulo, Brazil; 4 Faculdade de Medicina, Disciplina de Imunologia Clínica e Alergia (LIM-60), Universidade de São Paulo, São Paulo, Brazil; Centro Nacional de Microbiología - Instituto de Salud Carlos III, Spain

## Abstract

**Background:**

High genetic diversity at both inter- and intra-host level are hallmarks of RNA viruses due to the error-prone nature of their genome replication. Several groups have evaluated the extent of viral variability using different RNA virus deep sequencing methods. Although much of this effort has been dedicated to pathogens that cause chronic infections in humans, few studies investigated arthropod-borne, acute viral infections.

**Methods and Principal Findings:**

We deep sequenced the complete genome of ten DENV2 isolates from representative classical and severe cases sampled in a large outbreak in Brazil using two different approaches. Analysis of the consensus genomes confirmed the larger extent of the 2010 epidemic in comparison to a previous epidemic caused by the same viruses in another city two years before (genetic distance = 0.002 and 0.0008 respectively). Analysis of viral populations within the host revealed a high level of conservation. After excluding homopolymer regions of 454/Roche generated sequences, we found 10 to 44 variable sites per genome population at a frequency of >1%, resulting in very low intra-host genetic diversity. While up to 60% of all variable sites at intra-host level were non-synonymous changes, only 10% of inter-host variability resulted from non-synonymous mutations, indicative of purifying selection at the population level.

**Conclusions and Significance:**

Despite the error-prone nature of RNA-dependent RNA-polymerase, dengue viruses maintain low levels of intra-host variability.

## Introduction

**Figure 1:**
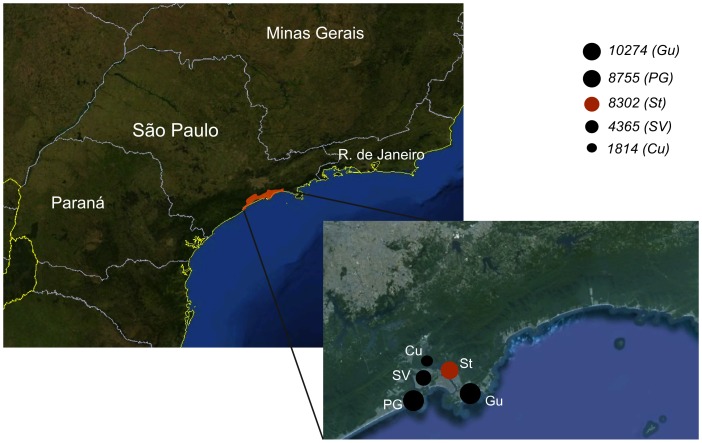
Geographic region of 2010 Santos epidemic. Southern São Paulo coastal region where viruses from this study were sampled are in red in the São Paulo map. Full black and red circles in the region focused represent the cities affected. The size of the circles specifies the magnitude of the epidemic in each city, also given by the number of reported cases in Santos (St), Praia Grande (PG), Guarujá (Gu), São Vicente (SV) and Cubatão (Cu).

Dengue is the most frequent arthropod-borne human viral infection in tropical and sub-tropical regions, with one hundred million people at risk annually. In the last two decades, Brazil has been responsible for more than 60% of the total reported dengue fever cases in the Americas, experiencing severe yearly outbreaks of serotypes 1 to 3 [1]. In 2008, serotype 4 re-emerged in the northern region of Brazil [2] and rapidly spread to other regions, causing small outbreaks in at least six different states. Today, the country experiences outbreaks of all four serotypes. For this study, samples were collected during a large outbreak in the State of São Paulo coastal region during the first semester of 2010, with more than 108,000 reported cases, including 81 deaths associated with the severe forms of the disease [3]. This epidemic affected a geographic area nearby the city of Santos, with the greatest number of cases reported in five nearby cities (Figure 1).

Dengue has limited intra-genotype diversity, which is modulated by two main processes: (i) the host’s immune response, which exerts a selective pressure on the virus, and (ii) the bottlenecks at transmission (two in this case, the vertebrate-invertebrate alternate cycle) [4]. As a result, a balance between variability gain due to mutations in each host and the maintenance of these mutations through natural selection is shaping the resulting viral diversity observed at the population level.

Our current understanding of dengue intra-host diversity has been mostly limited to the analysis of single genes of dengue serotypes 1 to 3 using conventional cloning and Sanger sequencing methods [5]–[7]. Most studies agree that the level of intra-host diversity is relatively high with mean pairwise differences reaching up to 1.67%. Recently, a study conducted by Thai *et al.* [8] assessed dengue diversity by sampling a larger number of clones while using more rigorous methods to validate the mutations and reported much lower intra-host variability estimates than previously reported (0.008%,).In the last decade, deep sequencing technologies have emerged capable of sequencing individual molecules directly from PCR amplicons without the need for cloning thereby enabling researchers to study viral *quasispecies* diversity with unprecedented resolution. Work by our group and others has employed 454 pyrosequencing, a method for deep sequencing, for whole-genome shotgun sequencing of RNA viruses causing chronic infections, such as hepatitis C virus (HCV), human immunodeficiency virus (HIV), and simian immunodeficiency virus (SIV) [9]–[14], but studies on the evolutionary dynamics of acute infecting viruses within individuals are limited [15]. Here, we employed pyrosequencing in combination with a transposon-based fragmentation method to capture inter and intra-host diversity of near full-length DENV-2 genomes covering the entire open reading frame (ORF) in representative samples obtained during the 2010 outbreak in cities located in the State of São Paulo coastline [3]. By sequencing viruses sampled from 10 infected individuals we were able to confirm that the level of intra-host viral variability is much lower than previously reported, reaching approximately 0.002%, and determined the level of variability throughout four months of the outbreak.

## Materials and Methods

### Ethics Statement

The current project was conducted after Hospital das Clínicas -University of São Paulo’s Institutional Review Board (CAPPesq) approval, under protocol #0652/09. Written informed consent was obtained from all participants. Written informed consent was also obtained for children through their parents.

### Patients and Samples

Serum samples were obtained from patients with clinically suspected dengue fever treated at the Ana Costa Hospital in Santos, coastal region in the State of Sao Paulo, Brazil, during the 2010 DENV2 epidemic [16]. Dengue infections were confirmed and serotypes were determined by using commercially available rapid tests (NS1, IgG, and IgM) as well as multiplex reverse transcriptase polymerase chain reaction (RT-PCR) [17]. Patients were selected to be representative of the outbreak. Therefore, samples were obtained from cases diagnosed from February through May, 2010, from either classic dengue fever cases and severe cases.

To determine the viral load, RNA was extracted from 140 µl of plasma using the Qiagen Viral RNA mini kit (Qiagen, Valencia, USA) according to the manufacturer’s instructions. RT-PCRs were performed in duplicate using SuperScript® III Platinum® SYBR® Green One-Step RT-qPCR with ROX kit (Invitrogen, Inc., EUA) and pan-dengue primers covering all 4 serotypes [18]. An internal control (Bovine Diarrhea Virus - BVDV, a flavivirus grown in cell culture) was added to the samples before extraction and also submitted to a parallel Real-time PCR assay. Supernatant from DENV-3 cell cultures was included as external control in every RT-qPCR run.

Several samples were also isolated in cell culture using the established C6/36 *Aedes albopictus* cell line (ATCC number CRL-1660). After the onset of a cytopathic effect (CPE) associated with DENV infection, supernatants were collected and subjected to RT-PCR in order to confirm the serotype. To discriminate between primary and secondary dengue virus infections, the antigen-binding avidity of specific IgG was measured using a modified Dengue ELISA IgG kit (Focus Technologies) [19].

### RNA Extraction and Deep-sequencing

500 µl of plasma from acutely ill patients were centrifuged at 3,000 rpm for 5 min to remove cellular debris followed by centrifugation at 14,000 rpm for 60 min to concentrate the virus. To evaluate if the viral population diversity found in plasma is maintained after isolation in insect cell culture, we isolated viruses from one acutely infected patient and sequenced the viruses (i) direct from plasma and (ii) after one-week culture in C6/36 cell line. Viral RNA was extracted from 200 µl of plasma or cell culture supernatant containing the concentrated virus using the QIAamp Viral RNA Mini Kit (Qiagen, Valencia, USA) according to the manufacturer’s instructions. Primers were designed to amplify the complete open reading frame with three to six overlapping PCR amplicons of approximately 2–4 kb (See Table S1 for primers sequences used), based on conserved regions identified after a multiple sequence alignment of complete DENV2 genomes of the American/Asian genotype. Viral RNA was reverse transcribed and amplified using the SuperScript III high-fidelity one-step reverse transcription-PCR (RT-PCR) kit (Invitrogen, Life Technologies, Carlsbad, CA) with the following conditions: 50°C for 30 min; 94°C for 2 min; 40 cycles of 94°C for 15 s, 55°C for 30 s, and 68°C for 2–4 min; and 68°C for 5 min. Following amplification, PCR amplicon bands were isolated using gel electrophoresis (1% agarose) and purified using a Qiagen MinElute gel extraction kit (Qiagen, Valencia, CA). Each amplicon was quantified using Quant-IT HS reagents (Invitrogen, Life Technologies, Carlsbad, CA), and all amplicons from a single viral genome were pooled together at equimolar ratios. Each pool was then quantitated, and approximately 50 ng of each was fragmented, using a Nextera DNA sample prep kit (Roche Titanium compatible; Epicentre Biotechnologies, Madison, WI) according to the manufacturer's protocol. Final libraries representing each genome were characterized for average size by use of a high-sensitivity DNA Bioanalyzer chip on a model 2100 Bioanalyzer (Agilent Technologies, Loveland, CO) and were quantified with Quant-IT HS reagents (Invitrogen, Life Technologies, Carlsbad, CA). Libraries were then subjected to emulsion PCR, and enriched DNA beads were loaded onto a picotiter plate and pyrosequenced with a Roche/454 GS Junior sequencer using titanium chemistry (454 Life Sciences, Branford, CT).

Pyrosequencing data were analyzed using CLC Genomics Workbench 5.5 (CLC Bio, Aarhus, Denmark). Initially, reads were trimmed to remove short and low-quality reads. Using a phylogenetically related DENV2 genome as reference (GenBank ID GU131864), reads were assembled and the resulting consensus sequences were then used as each subject’s sequence in a reference guided alignment to assess intra-host variability. The single nucleotide polymorphism (SNP) analysis was performed using CLC's SNP analysis tool, applying the following parameters: window length = 7, maximum gap and mismatch count = 2, minimum central quality base = 30, minimum average quality for window bases = 25, minimum coverage = ×100, and minimum variant frequency = 1% (defined as the percentage of nucleotides that differ from the reference). Parameters used in this analysis were chosen to include variants more likely to be biologically significant, based on previous studies that used Roche/454 data [12], [14], [20]. The 1% frequency cut-off both maximizes the chance of detecting minor variants while at the same time reducing the probability to detect errors generated in vitro [12], [14]. Becker and co-workers used this cut-off as well as cut-offs below 1% for comparison and showed that if the coverage is higher than 100×, variants found at 1% frequency are most likely real, and the lower the coverage is, the higher the probability of the variant to be an artifact [20]. Also, by choosing high-quality values for both the central base (the SNP base) and the window bases (bases surrounding the central SNP base, both upstream and downstream), as well as having a minimum coverage of ×100, only areas of high quality and high coverage were considered for SNP calling. To avoid erroneous base calls in homopolymer regions, we mapped all the homopolymers through the genome by searching for regions of three or more identical consecutive nucleotides, and the deletions and insertions (DIPs) and SNPs that mapped in these regions were dismissed from Roche/454 generated sequences due to the high probability of miscalls.

In addition to the samples sequenced in 454 Roche, one different plasma sample was sequenced on the Illumina MiSeq using direct sequencing as previously described [14]. Briefly, after isolation of RNA from cell-free plasma and DNase I treatment, double stranded DNA was generated using the Superscript double-stranded cDNA Synthesis kit (Invitrogen, Carlsbad, CA, USA) primed with random hexamers thereby omitting any amplification. Approximately 1 ng of cDNA was then fragmented using the Nextera DNA Sample Preparation kit (Illumina, San Diego, CA, USA), quantified and subjected to deep sequencing on the Illumina MiSeq. Sequencing data were analyzed applying the same parameters previously used for the pyrosequencing data using CLC Genomics Workbench 5.5.

### Sanger Sequencing

To compare the variability obtained by deep sequencing methods to traditional cloning and Sanger sequencing, the capsid gene and part of the prM gene of the sample ACS538 were subjected to traditional Sanger sequencing. Viral RNA was extracted as described above and single strand cDNA was generated with random hexamers using the High Capacity cDNA Reverse Transcription Kit (Invitrogen, Life Technologies, Carlsbad, CA) followed by amplification with the TaqPlatinum Hi Fidelity kit (Invitrogen, Life Technologies, Carlsbad, CA) using primers described in Table S1. The resulting 615-nucleotide (nt) fragment was cloned into the pCR-TOPO TA vector (Invitrogen, Life Technologies, Carlsbad, CA) and transformed into *E. coli* DH5alpha quimio-competent cells. Thirty-one clones were sequenced on the ABI3100 using Big-Dye7 Terminator (Applied Biosystems, Warrington,UK), with M13 forward and M13 reverse primers. Chromatograms were analyzed in CodonCode Aligner v.3.0 (available at http://www.codoncode.com/) with a Phred quality score of 20 as cut-off for trimming of low-quality sequences and manually aligned and inspected using SeAl (http://tree.bio.ed.ac.uk/software/seal/).

### Phylogenetic Analysis

Consensus complete genome sequences obtained by sequencing on the GS Junior and the MiSeq were aligned to globally sampled DENV2 genomes (See Table S2 for a description of the sequences used) using Muscle [21]. Bayesian phylogenetic trees were constructed for 124 DENV2 genomes using MrBayes v.3.1.2 [22]. Two independent runs of 50 million steps each were done using GTR with gamma-distributed rate variation substitution model and a proportion of invariant sites as suggested by Akaike’s information criterion (AICc) in jModeltest [23]. Sampled trees were summarized and the consensus tree was visualized in FigTree v1.3 (http://tree.bio.ed.ac.uk/). Selection pressures were evaluated by comparing the rate of non-synonymous changes per non-synonymous site (dN) to the rate of synonymous changes per synonymous sites (dS) inferred for entire polyprotein as well as for specific sites (by codon) of all the consensus genomes by single-likelihood ancestor counting (SLAC), using the open-source HyPhy package [24]. Genetic diversity was also estimated for the consensus complete genomes at both the nucleotide and amino acid levels using Mega 5 [25]. MacClade v4.08 was used to assess nucleotide and amino acid substitutions along the branches [26].

## Results

### Epidemiological and Evolutionary Patterns of Santos DENV2

American/Asian DENV-2 complete genomes consensus were reconstructed from overlapping RT-PCR amplicons deep sequenced on the Roche/454 GS Junior instrument. Additionally, one plasma sample from an individual with classical dengue was sequenced on the Illumina MiSeq by direct sequencing [27]. Therefore, viral complete genomes were generated from ten infected patients that manifested either classical fever (eight individuals) or severe dengue (two individuals), and also from one sample cultured in C6/36 cell line. Disease outcome was determined according to the WHO’s 2009 Dengue Guidelines [28], where three possible classifications were adopted: (i) Dengue without warning signals (classical), (ii) Dengue with warning signals (classical WS), and (iii) severe Dengue. Table 1 summarizes all samples used in this study as well as key clinical and laboratory relevant features. There was no evident correlation between age, gender or viral load and disease outcome. The complete genomes of all DENV2 viruses were submitted to GenBank, under IDs JX286516 to JX286526 (Table 1).

**Table 1:** Clinical and laboratorial information of DENV-2 samples.

Patient	GenBank ID	Age(years)	Gender	Clinicalmanifestation	Primary XSecondary	Platelets/ml	Samplingday*	VL	Samplingdate
ACS46	JX286516	55	Female	Classical	2^nd^	113000	1	2.8E6	03/01/2010
ACS46sn$	JX286517	–	–	–	–	–	–	–	–
ACS380*	JX286526	67	Female	Classical	2^nd^	124000	2	6.6E6	03/30/2010
ACS538	JX286518	62	Female	Severe	2^nd^	43000	3	9.2E6	04/10/2010
ACS542	JX286519	1	Male	Severe	ND	24000	3	4.5E6	04/12/2010
ACS721	JX286521	34	Female	Classical	ND	191000	4	1.18E7	05/04/2010
DGV34	JX286522	50	Male	Classical	2^nd^	158000	3	1.4E7	02/24/2010
DGV37	JX286520	46	Female	Classical	2^nd^	132000	2	8.9E5	02/24/2010
DGV69	JX286525	56	Male	Classical	2^nd^	122000	2	2.07E7	03/09/2010
DGV91	JX286523	45	Female	Classical	2^nd^	233000	3	1.2E6	03/24/2010
DGV106&	JX286524	82	Female	Classical	2^nd^	10000	4	4E5	04/15/2010

$ supernatant of sample ACS46 cultured viruses in C6/36 (1week).

& Although the low platelets number, the patient had no additional evidence of severity.

* Days after the onset.

VL- viral load in copy number/ml.

Nd- not done.

**Figure 2:**
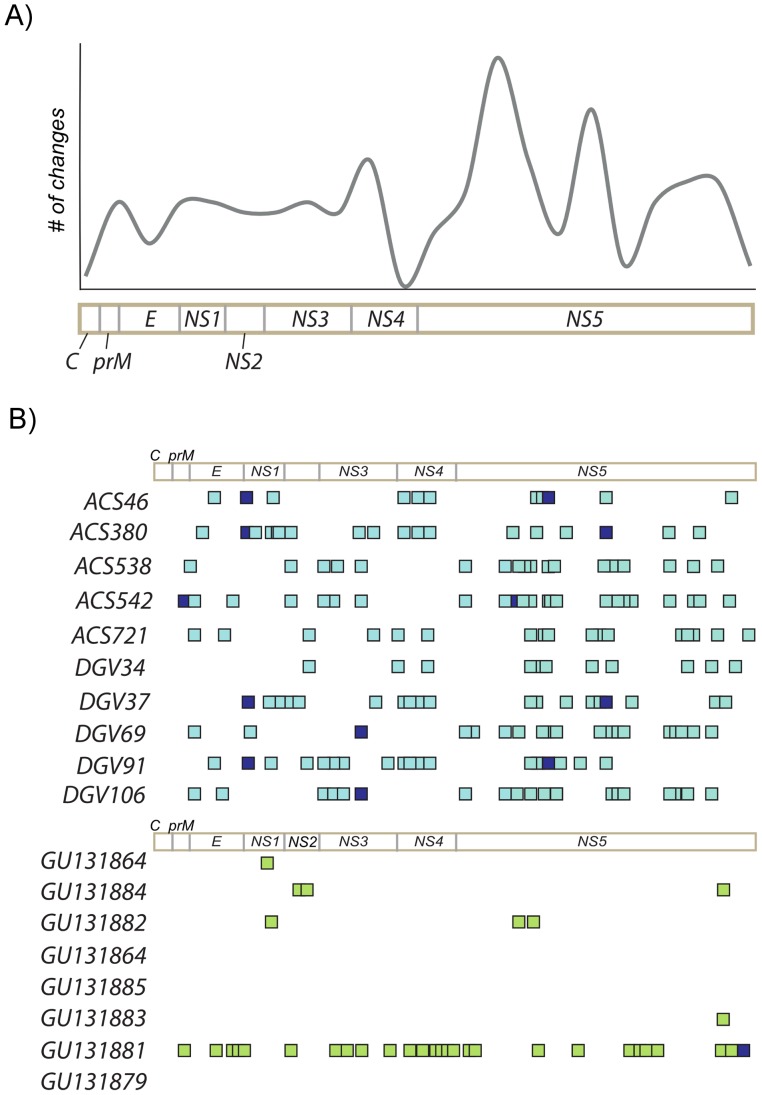
Inter-host variability. A- The line graph summarizes the level of accumulated variability per genome region across the Santos consensus viruses. B- Variability (synonymous changes) among consensus sequences sampled in Santos (light blue squares) compared to the variability among viruses from previous epidemics in 2008 in Ribeirão Preto, SP (green squares). Non-synonymous changes are represented in dark blue in both populations.

To determine the extent of DENV variability at a population level, we examined the consensus sequence of all viruses. We were not able to identify specific nucleotide or amino acid substitutions that distinguished classical and severe cases. Nucleotide pairwise distance calculated for all consensus genomes showed high degree of conservation, with a maximum distance observed between DGV37 and DGV69 (0.0044). Selection pressure analysis performed across the whole coding region and also by codon revealed no signs of positive selection (w = 0.03) as well as no codon under selection. The mean genetic diversity of the population (π) across the entire polyprotein was 0.002 for nucleotide and 0.0008 for amino acid sequence, indicative of protein conservation. Changes in 84 nucleotide sites were distributed throughout the coding region, and only nine were non-synonymous with three of them exclusively found in ACS542 (prM, NS3 and NS4a) (Figure 2B). Surprisingly, while the envelope gene exhibited low variability at inter-host level, the NS5 showed higher variability among samples (Figure 2A). It is also interesting to observe that the variability among DENV2 population sampled in this outbreak was much higher than that observed in viruses that circulated in a 2008 epidemic in Ribeirão Preto, a city located in the North of the State of São Paulo (represented by light green squares 2B), even though they belong to the same lineage.

### Phylogenetic Analysis

**Figure 3:**
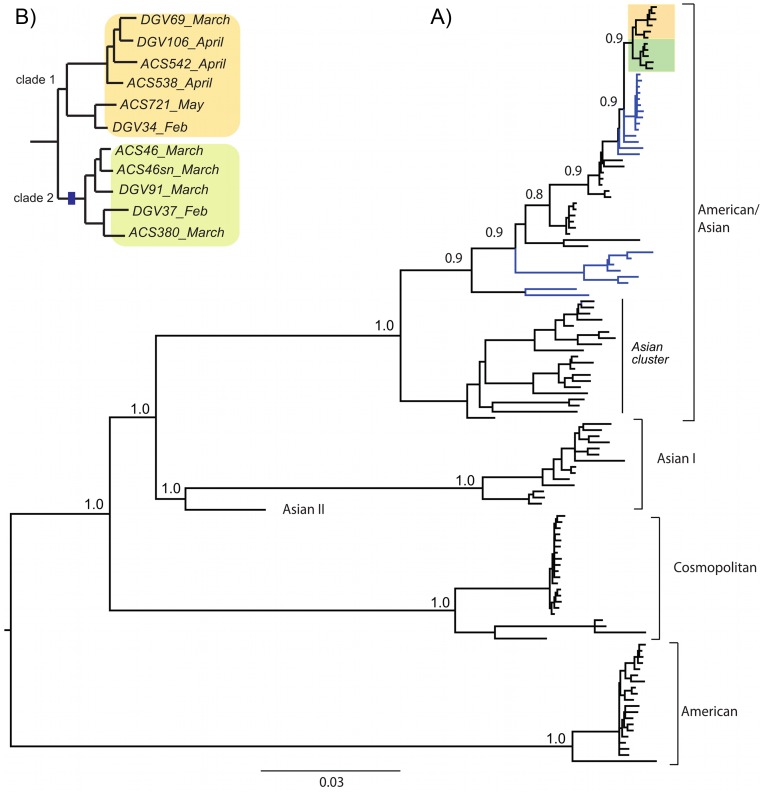
Bayesian phylogenetic tree of 124 DENV2 complete genomes. A-The tree shows the eleven Brazilian viruses sequenced in this study (two-color highlighted cluster at the top) and globally sampled DENV2 genomes. Blue branches represent Brazilian viruses sampled in previous epidemics. Posterior probability of all key nodes is depicted. B- Clusters of viruses sequenced during this study. The blue bar in the branch leading to clade 2 represents the amino acid change at position T180I in envelope gene.

To evaluate Dengue viruses in an epidemiological context, a Bayesian phylogenetic tree was constructed using 124 complete genomes comprising all five DENV-2 human genotypes and the eleven genomes sequenced from the 2010 DENV2 Santos outbreak (Figure 3A) (GenBank accession numbers are listed in table S2). All genomes sequenced in this study clustered with viruses that circulated in previous outbreaks registered in São Paulo and Rio de Janeiro States in 2008, but separated from those that circulated in older epidemics [16], [29]. Based on our results, Brazilian DENV viruses are not monophyletic, with viruses from Cuba, USA and the Dominican Republic clustering with those sequences.

The eleven 2010 DENV-2 genomes sequenced here segregated into two sub-populations, named clade 1 (DGV34, DGV69, DGV106, ACS538, ACS542 and ACS721) and clade 2 (DGV37, DGV91, ACS46p, ACS46sn and ACS380) (Figure 3B). By mapping all changes along the branches leading to them we found that viruses from clade 2 accumulated eleven nucleotides changes (eight C-T and two G-A transitions and one C-A transversion) in relation to the clade 1. One of them, a transition of cytosine to thymine at position 180 of the envelope gene led to an amino acid replacement (Thr to Ile). We also detected variability in NS1, NS3, and NS4b but found no evidence of association with virulence or pathogenicity.

### Deep Sequencing of DENV2 Genomes

On average, 44,000 reads per sample (±20.800) were generated with an average coverage of ∼1226 reads per nucleotide in Roche/454 (Table 2). Between 0.1 and 1.7% of the reads could not be mapped to Dengue genomes. Blast analysis revealed that 99% of the unspecific reads result from bacteria, while none of the remaining reads matched to the human genome. Less than 0.001% of the unmapped reads come from DENV.

**Table 2:** Deep Sequence obtained results and Inter-host viral variability.

Sample	Genome Sizesequenced	# sites excludingHPs	# Reads(x1000)	AverageCoverage/nt	# variable sites(SNP)	Variable sites/genome size (%)	Non-synonymousvariations (%)	# total of ntvariants	# of DIP	π
ACS46p	10598	8420	24.7	717	10	0.11	3 (30%)	257	7	4.2E-5
ACS46sn	10540	8362	27.5	818	14	0.16	5 (35%)	423	8	6.2E-5
ACS380*	10630	–	1.434	11.100	44	0.4	26 (59%)	12377	2	1.0E-4
ACS538	10544	8366	59.2	1628	15	0.17	9 (60%)	989	8	7.2E-5
ACS542	10540	8362	33	970	13	0.15	4 (30%)	413	3	4.0E-5
ACS721	10597	8419	45.5	1250	10	0.18	7 (70%)	42	4	4.0E-6
DGV34	10600	8422	48.7	1210	32	0.38	15 (45%)	353	0	3.5E-5
DGV37	10600	8422	91.3	2576	10	0.18	2 (20%)	373	2	1.7E-5
DGV69	10600	8422	33	913	14	0.16	6 (42%)	399	0	5.1E-5
DGV91	10611	8433	96.2	2186	34	0.4	20 (58%)	1068	0	5.8E-5
DGV106	10574	8396	109.7	2770	12	0.14	5 (42%)	1288	0	5.5E-5

All estimates were done after excluding homopolymer sites (HPs) from virus sequenced in 454.

The % *Variable sites/genome size* was calculated by dividing the *number of variable sites (SNP)* per *# sites excluding HPs*. The *“# total of nt variants*” does consider the total amount of nucleotides (reads) that vary in such position. This number was used to calculate the overall genetic variability.

Homopolymer regions can induce erroneous base calls on the Roche/454 platform, therefore all SNPs and DIPs that mapped to those regions were disregarded in subsequent analyses. Between 32 and 85 DIPs for each sample sequenced were recovered by 454/Roche and only two DIPs by Illumina. More than 90% of 454/Roche DIPs mapped to homopolymer regions and about 80% of them were identified as deletions in homopolymers of A, followed by a much lower proportion of Ts, Cs and Gs, respectively (Figure S1). The presence of DIPs was correlated to the size of the homopolymer and most DIPs occurred in regions of more than 5 nucleotides (Figure S1, right top graph). A total of 624 homopolymers (ranging from 3 to 6 consecutive bases) were found along the DENV2 genome, resulting in 2178 sites excluded from the analysis of intra-host genetic diversity (Table 2).

The level of intrahost variability obtained through Illumina direct sequencing was also evaluated for the sample ACS380 and was comparable to the observed through 454 pyrosequencing (Table 2, Tables S3 and S4).

### Sanger Sequencing of Capsid and prM

In parallel, the entire capsid and part of the prM gene of ACS538 (400 base pairs) was amplified using similar conditions and 31 clones were sequenced. The resulting 12378 nucleotides sequenced revealed twelve changes randomly distributed among the clones. One change was observed twice, but the remaining eleven were singletons. None of the changes were detected by deep sequencing and the unique variable site in the capsid recovered by deep sequencing was not observed in Sanger sequences probably due to the very low frequency (1.6%).

### Low Intra-host Genetic Diversity of DENV2

Overall, the two deep sequencing methods revealed between 10 and 44 variable sites per genome, resulting in approximately 0.002% of variability in all sequenced sites (Table 2 and see also Table S3 for details of frequency and coverage of all SNPs). DGV37, ACS46 and ACS721 had the highest conservation, with only 10 sites each exhibiting some variability and ACS380 had the lowest conservation, with 44 variable sites. The average viral load of the samples was around 7E+06 copies per ml, suggesting that the low level of diversity observed was not a result of low template abundance.

Among all 199 variable sites, 185 mapped into the coding region and 14 were found in either 5′UTR or 3′UTR. Among the sites that mapped to the polyprotein, 93 (50%) were in the first and second codon positions, resulting in non-synonymous changes. High proportions of non- synonymous mutations at the intra-host level have previously been reported for DENV1 [30] consistent with sequences evolving under or close to neutrality. In contrast, only 10% of all changes detected across the consensus sequences were non-synonymous, confirming that distinct selection pressures are imposed at short term and long term dengue evolution [30].

Variable sites were randomly distributed across the genes. Although we hypothesized that the envelope gene would accumulate the greatest number of viral variants due to immune pressure exerted by host immune system, our data did not support this hypothesis since only 13 sites exhibited some variability at envelope region (2.6%). Among the seven non-synonymous variants, four were into Domain II (EDII). With exception of I129F/V for DGV37 where variant phenylalanine was at a frequency of 6%, the remaining non-synonymous variations occurred in a frequency below 2% and none of them mapped into known immunogenic regions or were related to infection. Three non-sense changes were found (capside, NS3 and NS4b), but they appeared in a frequency below 2% in all cases (Table S4 and see also Table S3 for details of frequency and coverage of all SNPs.). Comparison of ACS46 sequenced directly from plasma to the same sample being cultured for one-week revealed similar levels of genetic variability (4.2 and 6.2×10^−5^ respectively) (Table 2). Although the consensus genomes were identical, variable sites within population were not the same. Only two synonymous variable sites were detected in both virus populations and a silent variant with a frequency of 36% in cell-isolated viruses was not detected at all in plasma population.

Despite such a low number of intra-host variability, particular sites showed some variability in distinct viral populations (Table S4). Among fourteen ‘shared’ variable positions, seven were non-synonymous, distributed through capsid, envelope, NS3 and NS5 and only the capsid 26 V was also variable at the inter-host level.

## Discussion

To date, within-host diversity of RNA viruses has been studied most comprehensively in HIV-1 and HCV [11]–[14], [31]. Here, we analyze intra and inter host diversity of dengue from patients infected with classical and severe forms of the disease that circulated during the same epidemic.

One of the challenges in studying viral diversity using massive parallel sequencing is the discrimination between *bona fide* and artificial variants since the type and rate of the error introduced by each platform is different. For example, insertion and deletion (DIPs) errors occur more frequently on the 454/Roche platform, especially in homopolymer regions, while the Illumina platform is more prone to mismatch errors [32], [33]. Furthermore, differences in the sample preparation, such as PCR-induced errors, could also potentially affect the results. Despite the 10 fold higher coverage obtained on the MiSeq platform, the number of variable sites or the total variability observed at the intra-host level was comparable between both methods. Interestingly, almost all DIPs detected by 454/Roche sequencing mapped to homopolymer regions suggesting that those variants were sequencing artifacts specific to this platform. Unfortunately, we did not have enough plasma from the nine individuals used for 454 sequencing to perform a head-to-head comparison.

### Intra-host Genetic Variability

Although the results described in this report are not based on large number of patients to carry out statistical analysis, some considerations can be discussed.

Despite the generally accepted high substitution rate of dengue viruses, estimated at 6.5×10^−4^s/s/y [34], [35], our data suggests that DENV-2 intra-host genetic variability is much lower than previously estimated for DENV serotypes 1 and 3, as determined by Sanger sequencing [5], [6], [36]. Nevertheless, because the very stringent parameters to validate variants obtained through deep sequencing, it is possible that we missed some *bona fide* mutations. Parameswaran *et al.,* also used deep sequencing to evaluate intra-host DENV2 variability in Nicaragua, but although they also noticed low intra-host variability, they observed 10 to 100× more variable sites across the genome compared to our results, with no evidences of mixed infection [37]. However, about 3 out 9 variable sites described by the authors as putative hotspots map to homopolymer regions and may be related to 454 errors.

By considering the number of reads with variability at any given site per total coverage at this site, the mean diversity of intra-host viral population was 4.8×10^−5^, which is consistent with previous results reporting DENV1 intra-host variability as 7.2×10^−5^ [8]. Interestingly, while Thai *et al*. used cloning and Sanger sequencing to evaluate variability, they also applied rigorous methods to validate variants eventually leading to results comparable to our study.

With few exceptions, most studies estimating DENV variability have used cloning and Sanger sequencing [5]–[8], [36]. In this study, we compared the variability obtained by deep sequencing to that recovered by cloning and Sanger sequencing. Although we are aware that an appropriate comparison is not possible here due to the low number of clones sequenced, the high number of changes observed in Sanger sequencing (∼ 0,1%) resembled previously reported results using the same method, but clearly contrasted to the results seen by deep sequencing here and by others [5], [7], [8], [37]. Importantly, Descloux and group evaluated the sequence variation produced by their in vitro system and found an error frequency of 0.1% [5], which is exactly the SNP frequency found here and in the previous studies that used Sanger.

Two SNPs found by Sanger (both from A to G) were also present in 454/Roche reads. However, they mapped in homopolymer regions of 3 and 4 Adenines and were in frequencies of 0.37 and 0.4% respectively (data not shown). Thus, according to the parameters adopted here to consider a variant to be true (i.e. 1% frequency, quality phred score above 30 and, most importantly, the exclusion of homopolymers), these variants were dismissed. We therefore suspect that much of the changes reported by studies that used Sanger sequencing without posterior validation of the variants represent artifacts generated during cDNA synthesis and PCR amplification [12], [38], which are more prone to be eliminated by deep sequencing analysis due to the high coverage and filter parameters. More rigorous experiments must be performed to evaluate how much of Sanger detected variants are due to experimental errors.

Arboviruses usually harbor less nucleotide and amino acid variation than observed in other RNA viruses due to alternate replication in mammalian and mosquito cells [39]–[41]. This alternation between hosts keeps arboviruses in high-fitness peaks that rarely overlap and only tolerate very few changes. In this scenario, genetically stable viruses would have evolutionary advantages over those that rapidly accumulate variants. In our case, the specialization of DENV to a single mammalian environment after urbanization may indeed have led to the selection of very specific genetic traits, further reducing optimal fitness peaks. Additional studies are needed however to determine how dengue restore adequate levels of diversity.

It is not unusual to use in-vitro cell culture to amplify viruses obtained from infected individuals prior to genetic and molecular studies [42], [43]. However, although one week of virus culture is probably not sufficient for the emergence of adaptive changes, the amount of variability that is gained or lost at the intra-population level during *in vitro* culture is largely unknown. To address this question, we compared deep sequenced viruses directly from human plasma to the same viruses after one-week of culture in C6/36 mosquito cells. As expected, the consensus sequences were identical, but the sites showing variability at the intra-host level were different. These findings indicate that different (low) selection pressures are exerted by C6/36 culture [44], which ultimately impacts on the generation of distinct viral populations. However, the influence of distinct selection pressure imposed by mammalian and invertebrate hosts on the generation of dengue diversity needs more investigation.

There is little (and controversial) evidence that intra-host genetic variability is linked to disease severity [5], [6], [37]. Although the small number of patients analyzed at a single time point presents a limitation to the present study, the amount of intra-host viral variability did not differ between classical and severe dengue cases. Subtle effects of specific variants on disease severity may not be detectable in such a small cohort and larger numbers must be evaluated to address this hypothesis.

Although only few sites exhibited variability in our study, some were consistently identified among distinct viral populations. Detection of the same variants in viruses circulating during the same epidemic suggests two possible and non-exclusive explanations: (*i*) common variable sites are a consequence of *de novo* mutations in less constrained sites (*i.e*. mutation hotspots). Mutation hotspots often reflect the mechanisms of generating mutations at a particular site (i.e. enzyme-induced hypermutation, homopolymers and repeat regions) [45], [46]. In viruses, hotspots are usually present in immunogenic sites and enable the escape of the host immune system [47], [48]. Nevertheless, shared variable sites in our sequences were not in homopolymer or repeat regions and also appeared in low frequency, suggesting that such mutations were non advantageous, at least in this context. And (*ii*), minor variants are not immediately eliminated by genetic drift and could be maintained during transmission to a new host. Although variable shared sites were found as minority population within the host, it has been demonstrated that minor variants can be passed between individuals regardless of their fitness, and sometimes for extended time-periods [36], [49]. Considering that the viruses sequenced in this study were sampled over a four months period, the maintenance of minor variants during this epidemic presents a realistic scenario.

### Genetic and Epidemiological Features of DENV2 from 2010 Epidemics

Our phylogeny, which includes globally sampled DENV genomes, confirms the Caribbean origin of the Brazilian American/Asian DENV2 strain [16], [29]. In fact, the presence of viruses from Central and North America (Cuba, Nicaragua, Guatemala, Jamaica, Dominican Republic and USA) within Brazilian sequences exemplifies the idea of continual exchange of viruses among countries on the American continent, and not only between Central and South America.

Interestingly, the separation of Santos viruses into two sub-clades and the high diversity found among the sequences in comparison to viruses sampled in previous epidemics (Ribeirao Preto, SP, Figure 2B) could be justified by the extent of both epidemics, where the number of cases reported in 2008 in Ribeirao Preto was around 1000 (http://www.cve.saude.sp.gov.br/htm/zoo/Den_gve08.htm) against more than 33 thousand cases reported in Santos coastal cities in a short five month period (http://www.cve.saude.sp.gov.br/htm/zoo/den09_import_autoc.htm). Although viruses sequenced in this study were all sampled in the city of Santos, it is a regional hub that is heavily trafficked by residents of neighboring cities.

In sum, the development of deep sequencing technologies provides new opportunities to study the viral diversity with unprecedented resolution. By using two different deep sequencing methods, we were able to describe, for the first time, the level of DENV2 variability intra and inter host throughout a large outbreak. The observed low level of variability may be a consequence of different pressures, including the constraints imposed by alternating hosts during virus evolution. Moreover, the putative intrinsically low rate of changes combined with the short time of viremia – common of acute infections – may exert less selective pressure on the virus than is commonly observed when studying chronic RNA virus infections.

## Supporting Information

Figure S1 **Deletions and Insertions (DIPs) sampled by Roche/454 GS Jr.** The main graph shows the absolute number of DIPs found per nucleotide obtained for the 10 viruses sequenced in Roche/454. The small graph at the top right specifies the proportion of DIPs in relation to homopolymer size.
(TIF)

Table S1 **Primers used to amplify the complete genome and also the capsid gene.**
(DOC)

Table S2 **GenBank accession numbers of Dengue viruses serotype 2 used to build the phylogenetic tree.**
(DOC)

Table S3 **Intra-host variability (SNPs). Reference positions are according to the ACS380 genome (JX286526), and the details of the amino acid changes are in Supplementary table 4.**
(DOC)

Table S4 **Variable non-synonymous sites at intra-host level.**
(DOC)
