# Supplementary figures and images for: Inter- and Intra-Host Viral Diversity in a Large Seasonal DENV2 Outbreak
Source: PLoS One. 2013 Aug 2;8(8):e70318. doi: 10.1371/journal.pone.0070318 (PMC3732279; doi:10.1371/journal.pone.0070318)

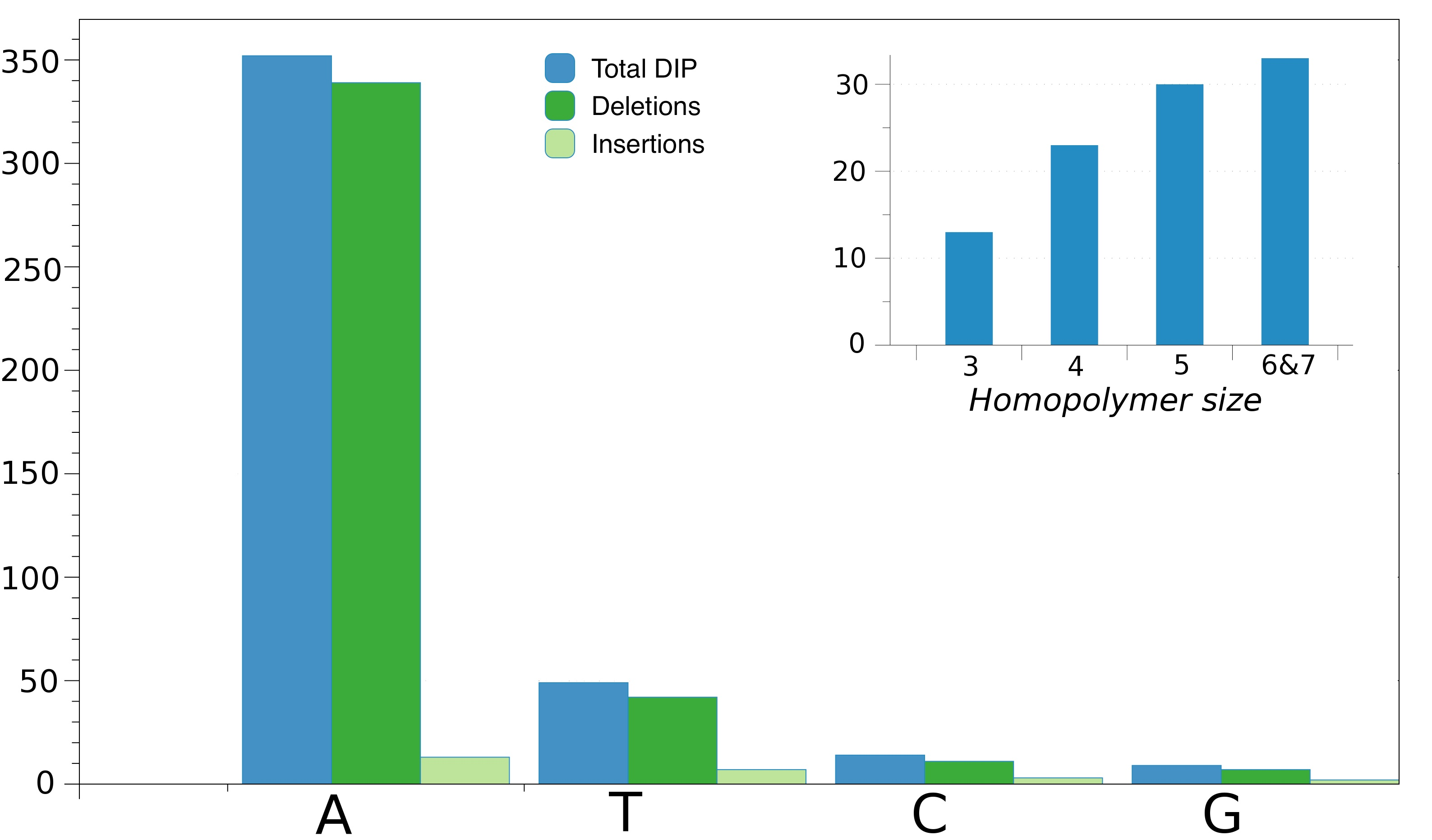

Supplement: Figure S1 — Deletions and Insertions (DIPs) sampled by Roche/454 GS Jr. The main graph shows the absolute number of DIPs found per nucleotide obtained for the 10 viruses sequenced in Roche/454. The small graph at the top right specifies the proportion of DIPs in relation to homopolymer size. (TIF) [file pone.0070318.s001.tif]
