# Supplementary material for: Inter- and Intra-Host Viral Diversity in a Large Seasonal DENV2 Outbreak
Source: PLoS One. 2013 Aug 2;8(8):e70318. doi: 10.1371/journal.pone.0070318 (PMC3732279; doi:10.1371/journal.pone.0070318)
Supplement: Table S1 — Primers used to amplify the complete genome and also the capsid gene. (DOC) [file pone.0070318.s002.doc]

Supplementary Table 1. Primers used to amplify the complete genome and also the capsid gene

| DENV2 – Primers for complete genome/capside | | |
| --- | --- | --- |
| Position | ID | Sequence |
| 28 | A1 FWD | GAC AGA TTC TTT GAG GGA GCT AA |
| 4120 | A1 REV | TGC TGG TTC TCG AAA GAG TTG |
| 3661 | A2 FWD | CGC TAC CAT GAC GGA TGA C |
| 7749 | A2 REV | TTT TGC TGA TCC TCG TGA CA |
| 6668 | A3 FWD | CAC AGA TAC AAC CAC ACT GG |
| 10644 | A3 REV | GGT CTT TCC CAG CGT CAA TA |
| *5355* | *A4 FWD* | *TCA CAG ACC CAG CAA GCA TA* |
| *7452* | *A4 REV* | *ACA CTG CAA TGG TGG TGT TC* |
| *7035* | *A5 FWD* | *AGG ATG GCC ATT GTC AAA GA* |
| *9133* | *A5 REV* | *GCG TAC ATT GCT CCT CCT TC* |
| *8496* | *A6 FWD* | *TAT GGT GAA CGG AGT GGT CA* |
| *10550* | *A6 REV* | *TCC TTC CAG TGA GAC TAC AGC TT* |
| 28 | D2_cap_FWD | TCTACGTGGACCGACAAAGAC |
| 623 | D2_623_REV | TGCACCAACAATCTATGTCTTC |

Italicized primers (A4 to A6) were alternatively used to cover regions not successfully amplified by A1, A2 and A3 primer pairs.
