# Supplementary material for: Inter- and Intra-Host Viral Diversity in a Large Seasonal DENV2 Outbreak
Source: PLoS One. 2013 Aug 2;8(8):e70318. doi: 10.1371/journal.pone.0070318 (PMC3732279; doi:10.1371/journal.pone.0070318)
Supplement: Table S2 — GenBank accession numbers of Dengue viruses serotype 2 used to build the phylogenetic tree. (DOC) [file pone.0070318.s003.doc]

**Supplementary Table 2**. GenBank accession numbers of Dengue viruses serotype 2 used to build the phylogenetic tree.

| **GenBank ID** | **Genotype** | **Country** |
| --- | --- | --- |
| JF327392 | Cosmopolitan | Singapore |
| JN851113 | Cosmopolitan | Singapore |
| JN851119 | Cosmopolitan | Singapore |
| JN851117 | Cosmopolitan | Singapore |
| DQ645556 | Cosmopolitan | Taiwan |
| DQ645554 | Cosmopolitan | Taiwan |
| DQ645552 | Cosmopolitan | Taiwan |
| DQ645550 | Cosmopolitan | Taiwan |
| DQ645547 | Cosmopolitan | Taiwan |
| DQ645548 | Cosmopolitan | Taiwan |
| DQ645546 | Cosmopolitan | Taiwan |
| DQ645553 | Cosmopolitan | Taiwan |
| DQ645551 | Cosmopolitan | Taiwan |
| DQ645549 | Cosmopolitan | Taiwan |
| DQ645545 | Cosmopolitan | Taiwan |
| DQ645555 | Cosmopolitan | Taiwan |
| DQ645544 | Cosmopolitan | Taiwan |
| DQ645543 | Cosmopolitan | Taiwan |
| DQ645540 | Cosmopolitan | Taiwan |
| DQ645542 | Cosmopolitan | Taiwan |
| DQ645541 | Cosmopolitan | Taiwan |
| HM582117 | American | Tonga |
| HM582116 | American | Tonga |
| HM582115 | American | Tonga |
| HM582113 | American | Tonga |
| HM582114 | American | Tonga |
| HM582111 | American | Tonga |
| HM582112 | American | Tonga |
| HM582105 | American | Samoa |
| HM582104 | American | Samoa |
| HM582106 | American | Samoa |
| HM582109 | American | French Polynesia |
| HM582108 | American | French Polynesia |
| HM582107 | American | Samoa |
| HM582110 | American | French Polynesia |
| HM582102 | American | New Caledonia |
| HM582100 | American | Fiji |
| HM582103 | American | New Caledonia |
| HM582101 | American | Fiji |
| HM582099 | American | Fiji |
| FJ898449 | American | Honduras |
| FM210246 | AsianI | Vietnam |
| FM210245 | AsianI | Vietnam |
| FM210216 | AsianI | Vietnam |
| EU482643 | AsianI | Vietnam |
| FM210242 | AsianI | Vietnam |
| FM210240 | AsianI | Vietnam |
| FJ410215 | AsianI | Vietnam |
| FJ687437 | AsianI | Thailand |
| FJ639832 | AsianI | Thailand |
| DQ181797 | AsianI | Thailand |
| GU131886 | AsianI | Thailand |
| AF169687 | AsianI | Thailand |
| AF169688 | AsianI | Thailand |
| AF169681 | AsianI | Thailand |
| M29095 | AsianII | New Guinea |
| FM210244 | American/Asian | Vietnam |
| FM210217 | American/Asian | Vietnam |
| FM210234 | American/Asian | Vietnam |
| FM210238 | American/Asian | Vietnam |
| FM210230 | American/Asian | Vietnam |
| FM210223 | American/Asian | Vietnam |
| FM210219 | American/Asian | Vietnam |
| FM210235 | American/Asian | Vietnam |
| FM210237 | American/Asian | Vietnam |
| FM210224 | American/Asian | Vietnam |
| FM210225 | American/Asian | Vietnam |
| FM210221 | American/Asian | Vietnam |
| FM210220 | American/Asian | Vietnam |
| FM21020 | American/Asian | Vietnam |
| FM210218 | American/Asian | Vietnam |
| FM210227 | American/Asian | Vietnam |
| FJ639701 | American/Asian | Cambodia |
| FJ639698 | American/Asian | Cambodia |
| FJ639703 | American/Asian | Cambodia |
| AF119661 | American/Asian | China |
| FJ850072 | American/Asian | Brazil |
| FJ850085 | American/Asian | Brazil |
| FJ850082 | American/Asian | Brazil |
| FJ850082 | American/Asian | Brazil |
| FJ850082 | American/Asian | Brazil |
| FJ850082 | American/Asian | Brazil |
| FJ850082 | American/Asian | Brazil |
| HQ026763 | American/Asian | Brazil |
| GU131864 | American/Asian | Brazil |
| GU131882 | American/Asian | Brazil |
| GU131880 | American/Asian | Brazil |
| GU131885 | American/Asian | Brazil |
| GU131879 | American/Asian | Brazil |
| GQ868549 | American/Asian | Brazil |
| GQ868551 | American/Asian | Brazil |
| HM181971 | American/Asian | Brazil |
| GU131884 | American/Asian | Brazil |
| GU131883 | American/Asian | Brazil |
| GU131881 | American/Asian | Brazil |
| GQ199890 | American/Asian | Brazil |
| GQ199892 | American/Asian | Jamaica |
| FJ898453 | American/Asian | Central America |
| EU687216 | American/Asian | Central America |
| AB122021 | American/Asian | Dominican Republic |
| AB122022 | American/Asian | Dominican Republic |
| AB122020 | American/Asian | Dominican Republic |
| EU687217 | American/Asian | Central America |
| EU482731 | American/Asian | Central America |
| AY702038 | American/Asian | Cuba |
| AY702037 | American/Asian | Cuba |
| AY702036 | American/Asian | Cuba |
| AY702039 | American/Asian | Cuba |
| AY702035 | American/Asian | Cuba |
| AY702034 | American/Asian | Cuba |
| AF489932 | American/Asian | Brazil |
| HQ999999 | American/Asian | Guatemala |
| GQ199893 | American/Asian | Mexico |
| JX286516 | American/Asian | Brazil* |
| JX286517 | American/Asian | Brazil* |
| JX286526 | American/Asian | Brazil* |
| JX286518 | American/Asian | Brazil* |
| JX286519 | American/Asian | Brazil* |
| JX286521 | American/Asian | Brazil* |
| JX286522 | American/Asian | Brazil* |
| JX286520 | American/Asian | Brazil* |
| JX286525 | American/Asian | Brazil* |
| JX286523 | American/Asian | Brazil* |
| JX286524 | American/Asian | Brazil* |

* This study
