# Supplementary material for: Inter- and Intra-Host Viral Diversity in a Large Seasonal DENV2 Outbreak
Source: PLoS One. 2013 Aug 2;8(8):e70318. doi: 10.1371/journal.pone.0070318 (PMC3732279; doi:10.1371/journal.pone.0070318)
Supplement: Table S3 — Intra-host variability (SNPs). Reference positions are according to the ACS380 genome (JX286526), and the details of the amino acid changes are in Supplementary table 4. (DOC) [file pone.0070318.s004.doc]

**Supplementary Table 3**

|  |  | **DGV37** |  |  |
| --- | --- | --- | --- | --- |
| Reference Position | Allele Variations | Frequencies | Coverage | Change in Protein |
| 102 | T/C | 98.8/1.2 | 251 |  |
| 182 | T/C | 98.8/1.2 | 806 | 26 cap |
| 1330 | A/T/G | 63/35.9/1.1 | 264 | 129 env |
| 1971 | G/A | 98.9/1.1 | 597 |  |
| 3669 | T/C | 98.7/1.3 | 1932 |  |
| 4863 | G/A | 98.1/1.9 | 1658 |  |
| 5328 | T/C | 98.9/1.1 | 1789 |  |
| 7041 | T/C | 96.0/3.9 | 3733 |  |
| 7299 | G/A | 95.2/4.8 | 2862 |  |
| 10538 | G/A | 99.0/1.0 | 191 |  |

|  |  | **DGV34** |  |  |
| --- | --- | --- | --- | --- |
| Reference Position | Allele Variations | Frequencies | Coverage | Change in Protein |
| 106 | A/G | 99.0/1.0 | 195 | 1 cap |
| 167 | A/G | 98.7/1.3 | 308 | 21 cap |
| 660 | G/A | 97.6/2.4 | 1915 |  |
| 1572 | C/T | 99.0/1.0 | 1891 |  |
| 2871 | C/T | 98.5/1.5 | 663 |  |
| 3627 | C/T | 99/1.0 | 1328 |  |
| 3631 | T/C | 96.8/3.2 | 762 |  |
| 4089 | T/C | 97.6/2.4 | 1304 |  |
| 4582 | T/C | 97.9/2.1 | 958 |  |
| 4998 | A/G | 98.3/1.7 | 239 |  |
| 5297 | T/C | 98.9/1.1 | 359 | 256 NS3 |
| 5715 | T/C | 62.5/37.5 | 328 |  |
| 5794 | G/A | 98.7/1.3 | 232 | 422 NS3 |
| 5898 | A/G | 98.3/1.7 | 181 |  |
| 6378 | A/G | 98.4/1.1 | 187 |  |
| 7166 | T/C | 98.6/1.2 | 429 | 111 NS4b |
| 7177 | G/A | 98.5/1.5 | 683 | 115 NS4b |
| 7317 | C/T | 97.9/2.1 | 815 |  |
| 7987 | C/T | 98.9/1.1 | 186 | 137 NS5 |
| 8773 | T/C | 98.9/1.1 | 263 | 399 NS5 |
| 8829 | C/T | 98.7/1.3 | 151 |  |
| 8887 | A/G | 98.0/2.0 | 102 | 437 SN5 |
| 9107 | A/G | 98.8/1.3 | 320 | 510 NS5 |
| 9501 | C/T | 97.4/2.6 | 234 |  |
| 9623 | A/T | 97.2/2.8 | 145 | 682 NS5 |
| 9628 | A/G | 97.9/2.1 | 140 | 684 NS5 |
| 9656 | A/G | 98.9/1.1 | 183 | 695 NS5 |
| 9692 | A/G | 98.0/2.0 | 197 |  |
| 9874 | T/C | 98.5/1.5 | 206 | 766 NS5 |
| 10197 | T/C | 98.8/1.2 | 161 |  |
| 10406 | A/G | 98.8/1.2 | 163 |  |
| 10416 | C/T | 98.5/1.5 | 135 |  |

|  |  | **ACS46_p** |  |  |
| --- | --- | --- | --- | --- |
| Reference Position | Allele Variations | Frequencies | Coverage | Change in Protein |
| 182 | T/C | 99.0/1.0 | 197 | 26 cap |
| 263 | T/C | 98.5/1.5 | 200 | 53 cap |
| 2895 | T/C | 98.7/1.3 | 533 |  |
| 5032 | A/G | 97.2/2.8 | 358 |  |
| 5259 | A/G | 98.4/1.6 | 253 |  |
| 5392 | T/C | 98.4/1.1 | 182 | 288 NS3 |
| 6291 | A/G | 97.2/2.8 | 106 |  |
| 7968 | C/T | 98.8/1.2 | 324 |  |
| 9215 | T/C | 98.9/1.1 | 283 |  |
| 9957 | A/G | 58.8/41.2 | 563 |  |

|  |  | **ACS46_SN** |  |  |
| --- | --- | --- | --- | --- |
| Reference Position | Allele Variations | Frequencies | Coverage | Change in Protein |
| 139 | C/T | 98.3/1.7 | 118 | 12 cap |
| 888 | A/G | 99/1 | 1176 |  |
| 1316 | A/G | 98.9/1.1 | 529 | 124 env |
| 2463 | A/G | 98.3/1.1 | 181 |  |
| *2895* | T/C | 98.3/1.6 | 863 |  |
| 4938 | A/G | 98.9/1.1 | 531 |  |
| 5419 | A/G | 98.8/1.2 | 164 | 297 NS3 |
| 5539 | A/G | 98.3/1.7 | 118 | 337 NS3 |
| 5615 | T/C | 98.8/1.2 | 172 | 362 NS3 |
| 7311 | C/A | 63.4/36.6 | 483 |  |
| 8149 | A/G | 96/4.0 | 382 |  |
| *9957* | A/G | 69.5/30.5 | 682 |  |
| 9213 | A/G | 94.5/5.5 | 333 |  |
| 10385 | A/G | 98.2/1.8 | 198 |  |
|  |  |  |  |  |
|  |  | **ACS721** |  |  |
| Reference Position | Allele Variations | Frequencies | Coverage | Change in Protein |
| 145 | A/G | 99.0/1.0 | 199 | 14 cap |
| 543 | C/T | 98.9/1.1 | 1015 |  |
| 1058 | A/G | 98.4/1.6 | 126 | 38 env |
| 1980 | A/G | 98.4/1.6 | 129 |  |
| 4523 | A/G | 99.0/1.0 | 486 | 127 NS2b |
| 4998 | A/G | 98.6/1.4 | 585 |  |
| 5128 | A/G | 97.8/2.2 | 134 | 200 NS3 |
| 8819 | C/T | 98.4/1.6 | 496 | 414 NS5 |
| 9218 | T/C | 98.9/1.1 | 267 | 547 NS5 |
| 9623 | A/T | 98.7/1.0 | 667 | 682 NS5 |

|  |  | **DGV106** |  |  |
| --- | --- | --- | --- | --- |
| Reference Position | Allele Variations | Frequencies | Coverage | Change in Protein |
| 182 | G/A | 98.9/1.1 | 638 | cap 26 |
| 588 | T/C | 52.1/47.9 | 2179 |  |
| 2471 | A/G | 98.5/1.5 | 135 | NS1 14 |
| 3370 | A/G | 98.6/1.4 | 437 | ns1 214 |
| 5008 | A/G | 98.8/1.2 | 1613 |  |
| 5128 | A/G | 98.5/1.5 | 332 | NS3 200 |
| 6339 | C/T | 98.8/1.2 | 1715 |  |
| 6420 | G/A | 99.0/1.0 | 2017 |  |
| 7467 | G/A | 98.8/1.2 | 683 |  |
| 7821 | T/C | 98.5/1.5 | 1241 |  |
| 7888 | A/G | 98.9/1.1 | 714 | NS5 104 |
| 8910 | A/G | 81.2/18.8 | 782 |  |

|  |  | **DGV91** |  |  |
| --- | --- | --- | --- | --- |
| Reference Position | Allele Variations | Frequencies | Coverage | Change in Protein |
| 205 | T/C | 99.0/1.0 | 398 | 34 cap |
| 266 | T/C | 99.0/1.0 | 298 | 54 cap |
| 336 | T/C | 96.5/3.5 | 373 |  |
| 1058 | A/G | 98.1/1.9 | 104 | 38 env |
| 2140 | G/A | 98.9/1.1 | 366 | 399 env |
| 2231 | T/C | 98.9/1.1 | 273 | 429 env |
| 2277 | T/C | 93.5/6.5 | 325 |  |
| 2936 | T/C | 98.1/1.9 | 154 | 169 NS1 |
| 3066 | G/A | 98.6/1.4 | 222 | 212 NS1 |
| 3260 | T/C | 98.6/1.4 | 216 | 277 NS1 |
| 3668 | G/C/A | 95.9/1.8/1.7 | 1038 | 61 NS2a |
| 4062 | A/G | 98.5/1.4 | 8960 | 192 NS2a |
| 4064 | C/T | 98.7/1.3 | 9031 | 193 NS2a |
| 4101 | T/C | 98.6/1.4 | 1913 |  |
| 4998 | A/G | 96.0/4.0 | 593 |  |
| 5032 | A/G | 98.6/1.4 | 222 | 168 NS3 |
| 5083 | A/G | 99.0/1.0 | 110 | 185 NS3 |
| 5462 | G/A | 97.5/1.7 | 118 | 311 NS3 |
| 5598 | G/A | 97.2/2.8 | 177 |  |
| 5614 | G/A | 99.0/1.0 | 200 | 362 NS3 |
| 5639 | A/G | 98.6/1.4 | 437 | 370 NS3 |
| 6258 | T/C | 92.5/7.5 | 294 |  |
| 6844 | G/A | 98.5/1.5 | 135 | 4 NS4b |
| 6888 | T/C | 63.2/36.8 | 1009 |  |
| 7155 | T/C | 98.0/2.0 | 2200 |  |
| 7695 | C/T | 98.0/2.0 | 1382 |  |
| 7869 | A/G | 99.0/1.0 | 1397 |  |
| 8712 | A/T | 99.0/1.0 | 1390 |  |
| 9354 | C/T | 98.7/1.3 | 1490 |  |
| 9359 | C/T | 98.2/1.8 | 1077 | NS5 594 |
| 9623 | A/T | 98.1/1.8 | 857 | 682 ns5 |
| 9650 | T/C | 93.4/6.6 | 1261 | 691 NS5 |
| 9981 | T/C | 98.6/1.4 | 1636 |  |
| 10471 | A/G | 98.7/1.3 | 224 |  |

|  |  | **DGV69** |  |  |
| --- | --- | --- | --- | --- |
| Reference Position | Allele Variations | Frequencies | Coverage | Change in Protein |
| 2996 | A/G | 98.7/1.3 | 301 |  |
| 4751 | A/G | 98.8/1.0 | 492 |  |
| 5920 | A/G | 98.6/1.0 | 293 | 464 NS3 |
| 6845 | T/C | 99.0/1.0 | 198 |  |
| 6847 | T/A | 98.5/1.5 | 135 | 5 NS4b |
| 7070 | G/A | 95.0/5.0 | 844 |  |
| 8798 | T/A | 52.4/47.6 | 656 |  |
| 8886 | A/G | 98.1/1.6 | 375 | 437 NS5 |
| 9032 | G/A | 99.0/1.0 | 387 |  |
| 9454 | A/G | 98.6/1.4 | 280 | 626 NS5 |
| 9575 | T/C | 99.0/1.0 | 289 |  |
| 9622 | A/T | 98.4/1.2 | 248 | 682 NS5 |
| 9846 | T/C | 98.7/1.3 | 289 | 757 NS5 |
| 10413 | A/G | 98.7/1.3 | 297 |  |

|  |  | **ACS538** |  |  |
| --- | --- | --- | --- | --- |
| Reference Position | Allele Variations | Frequencies | Coverage | Change in Protein |
| 167 | A/G | 98.4/1.6 | 123 | 21 cap |
| 615 | C/T | 50.6/49.4 | 788 |  |
| 1330 | A/T | 96.5/2.9 | 173 | 129 env |
| 1971 | G/A | 98.7/1.3 | 616 |  |
| 4051 | G/A | 75.7/24.3 | 407 | 189 NS2a |
| 5215 | G/T | 95.9/4.1 | 749 | 229 NS3 |
| 5259 | A/G | 97.4/2.6 | 306 |  |
| 5269 | A/C | 98.9/1.1 | 527 | 247 NS3 |
| 5279 | T/A | 51.4/48.3 | 617 | 250 NS3 |
| 5495 | G/C | 98.8/1.2 | 168 | 322 NS3 |
| 5539 | A/G | 98.5/1.5 | 134 | 337 NS3 |
| 5584 | A/G | 98.9/1.1 | 277 | 352 NS3 |
| 7785 | C/T | 91.1/8.9 | 609 |  |
| 9108 | A/G | 93.3/6.7 | 1001 |  |
| 9834 | T/C | 98.8/1.2 | 991 |  |

|  |  | **ACS542** |  |  |
| --- | --- | --- | --- | --- |
| Reference Position | Allele Variations | Frequencies | Coverage | Change in Protein |
| 270 | T/C | 98.9/1.1 | 179 |  |
| 1553 | A/C | 98.7/1.2 | 1368 | 203 env |
| 2740 | T/C | 96.8/3.2 | 1083 |  |
| 5128 | A/G | 99.0/1.0 | 193 | 200 NS3 |
| 5259 | A/G | 97.9/2.1 | 468 |  |
| 7589 | A/G | 98.8/1.2 | 245 | 4 NS5 |
| 7707 | A/G | 98.1/1.9 | 212 |  |
| 7806 | G/A | 98.8/1.3 | 160 |  |
| 7884 | C/T | 98.8/1.2 | 161 |  |
| 8010 | A/G | 96.3/3.7 | 162 |  |
| 8220 | C/T | 99.0/1.0 | 199 |  |
| 8230 | A/G | 98.9/1.1 | 184 | 217 NS5 |
| 9288 | G/A | 58.6/41.4 | 785 |  |

|  |  | **ACS380** | |  |
| --- | --- | --- | --- | --- |
| Reference Position | Allele Variations | Frequencies | Coverage | Change in Protein |
| 19 | T/A | 83.1/16.9 | 569 |  |
| 21 | T/C | 95.8/4.2 | 589 |  |
| 26 | G/A | 95.9/4.1 | 606 |  |
| 30 | C/T | 96.0/4.0 | 628 |  |
| 31 | G/A | 97.4/2.6 | 968 |  |
| 50 | A/T | 97.9/2.0 | 1738 |  |
| 72 | T/A | 85.2/14.8 | 3518 |  |
| 95 | G/T | 98.7/1.3 | 5517 |  |
| 97 | A/T | 98.7/1.3 | 5493 |  |
| 99 | A/C | 98.8/1.2 | 6156 |  |
| 105 | G/A | 98.7/1.3 | 7839 |  |
| 1809 | A/T | 98.6/1.4 | 10579 | 228 ENV |
| 2219 | T/A | 98.4/1.6 | 5893 | 425 ENV |
| 2662 | A/T | 97.6/2.4 | 9980 | 78 NS1 |
| 3402 | A/T | 99.0/1.0 | 9371 | 324 NS1 |
| 3736 | A/G | 98.8/1.2 | 8721 | 83 NS2a |
| 3738 | A/T | 98.7/1.3 | 8639 | 85 NS2a |
| 3740 | C/T | 98.7/1.3 | 8363 | 86 NS2a |
| 4387 | A/T | 97.5/2.5 | 12495 | 83 NS2b |
| 4489 | A/C | 98.9/1.1 | 13749 |  |
| 4671 | T/C | 98.5/1.5 | 13615 |  |
| 5313 | T/G | 98.5/1.5 | 10810 | 261NS3 |
| 5318 | C/A | 98.7/1.3 | 10751 | 263 NS3 |
| 5430 | T/C | 98.9/1.1 | 8151 |  |
| 5433 | A/T | 98.9/1.1 | 7818 |  |
| 5848 | C/T | 98.7/1.3 | 9667 | 440 NS3 |
| 5882 | A/G | 97.5/2.5 | 9724 | 451 NS3 |
| 5896 | C/T | 98.9/1.0 | 11667 | 456 NS3 |
| 6764 | G/A | 98.7/1.2 | 6047 | 127 NS4a |
| 6767 | C/A | 98.6/1.4 | 5829 | 1 2K |
| 6991 | C/T | 98.4/1.6 | 9760 | 53 NS4b |
| 7649 | A/C | 98.6/1.3 | 14299 | 24 NS5 |
| 7661 | A/C | 98.5/1.4 | 15219 | 28 NS5 |
| 7772 | T/C | 98.8/1.2 | 13447 | 65 NS5 |
| 8315 | A/C | 98.3/1.7 | 11545 | 246 NS5 |
| 8765 | G/A | 98.8/1.2 | 15202 | 396 NS5 |
| 8908 | G/C | 97.9/2.1 | 22028 | 444 NS5 |
| 9792 | C/A | 98.9/1.1 | 12076 |  |
| 10024 | G/T | 98.4/1.6 | 16650 | 816 NS5 |
| 10197 | T/C | 98.3/1.7 | 10925 |  |
| 10200 | A/T | 98.3/1.7 | 10737 |  |
| 10202 | G/C | 98.2/1.7 | 10685 | 875 NS5 |
| 10204 | A/T | 98.0/2.0 | 9222 | 876 NS5 |
| 10207 | G/A | 98.1/1.9 | 9442 | 877NS5 |
