# Supplementary material for: Inter- and Intra-Host Viral Diversity in a Large Seasonal DENV2 Outbreak
Source: PLoS One. 2013 Aug 2;8(8):e70318. doi: 10.1371/journal.pone.0070318 (PMC3732279; doi:10.1371/journal.pone.0070318)
Supplement: Table S4 — Variable non-synonymous sites at intra-host level. (DOC) [file pone.0070318.s005.doc]

| **Region** |  |  |  |  | VIRUS |  |  |  |  |  |  |
| --- | --- | --- | --- | --- | --- | --- | --- | --- | --- | --- | --- |
|  | DGV34 | DGV37 | DGV69 | ACS46 | ACS46sn | ACS721 | DGV106 | DGV538 | DGV542 | DGV91 | ACS380** |
| capsid | 1 (M-ns); **21 (N-S)** | **26 (V-A)** |  | **26 (V-A);** 53 (F-S) | 12 (P-S) | 14 (N-D) | 25 (T-A) | **21 (N-S)** |  | 34 (S-P); 54 (L-P) |  |
|  |  |  |  |  |  |  |  |  |  |  |  |
|  |  |  |  |  |  |  |  |  |  |  |  |
| protM |  |  |  |  |  |  |  |  |  |  |  |
|  |  |  |  |  |  |  |  |  |  |  |  |
| Env |  | **129 (I-F/V)** |  |  | 124(N-S) | 38 (K-R); |  | **129 (I-F/A)** | 203 (D-A) | 38 (K-R); 399 (G-S) | 228(R-S); 425 (L-Q) |
|  |  |  |  |  |  |  |  |  |  | 429 (F-S) |  |
|  |  |  |  |  |  |  |  |  |  |  |  |
|  |  |  |  |  |  |  |  |  |  |  |  |
| NS1 |  |  |  |  |  |  | 14 (K-R); 214 (K-E) |  |  | 169 (L-P); 212 (M-I) | 78 (I-F); 324(R-S) |
|  |  |  |  |  |  |  |  |  |  | 277 (F-S) |  |
|  |  |  |  |  |  |  |  |  |  |  |  |
| NS2a |  |  |  |  |  |  |  | 189(A-T)HP |  | 61 (G-A/D) | 83(R-G); 85(R-S) |
|  |  |  |  |  |  |  |  |  |  | 192 (I-M); 193 (P-L) | 86(P-L) |
|  |  |  |  |  |  |  |  |  |  |  |  |
| NS2b |  |  |  |  |  | 127(K-R) |  |  |  |  | 83(S-C) |
|  |  |  |  |  |  |  |  |  |  |  |  |
|  |  |  |  |  |  |  |  |  |  |  |  |
| NS3 | 256(I-T); 422 (V-I) |  | 464 (N-S) | 288(F-H) | 297(R-G); **337(R-G)** | **200(T-A)** | **200 (T-A)** | 229(A-S); 247(I-L) | **200(T-A)** | 168 (T-A); 185 (K-E) | 261(C-W); 263(A-D) |
|  |  |  |  |  | **362 (V-A)** |  |  | 250(V-E); 322(R-T) |  | 311 (G-E); **362 (V-I)** | 440(R-W); 451(S-R) |
|  |  |  |  |  |  |  |  | **337 (R-G);** 352(T-A) |  | 370 (D-G) | 456(G-stop) |
|  |  |  |  |  |  |  |  |  |  |  |  |
| NS4a |  |  |  |  |  |  |  |  |  |  | 127(R-K) |
|  |  |  |  |  |  |  |  |  |  |  |  |
| 2k |  |  |  |  |  |  |  |  |  |  | 1(T-K) |
| NS4b | 111 (L-P); 115 (V-I) |  | 5 (F-I) |  |  |  |  |  |  | 4 (G-S) | 53 (R-stop) |
|  |  |  |  |  |  |  |  |  |  |  |  |
| NS5 | 137 (P-S); 399 (F-L) |  | **437(R-G);** |  |  | 414(T-I) | 104 (T-A) |  | 4 (N-S); | 594 (S-L); **682 (N-I)** | 24(E-A); 28(Y-S) |
|  | **437(R-G);** 510(E-G) |  |  |  |  | 547 (L-S); **682 (N-I)** |  |  | 217(M-V) | 691 (I-T) | 65(F-S); 246 (K-T) |
|  | **682 (N-I);** 684 (M-V) |  | 626(E-G); **682 (N-I)** |  |  |  |  |  |  |  | 396(R-K); 444 (E-Q) |
|  | 695(E-R); 766 (Y-S) |  | 757 (S-P); |  |  |  |  |  |  |  | 816 (V-L); 875(G-A) |
|  |  |  |  |  |  |  |  |  |  |  | 876(D-Y); 877(E-K) |
|  |  |  |  |  |  |  |  |  |  |  |  |

**Supplementary Table 4. Variable non-synonymous sites at intra-host level.**

- **Sites in bold were found in different samples**
- **** ACS380 was sequenced by RNA seq in Illumina**
